# Network analysis of social support, resilience, quality of life, and insomnia under pandemic control measures

**DOI:** 10.3389/fpubh.2026.1801742

**Published:** 2026-05-14

**Authors:** Min Dai, Shuyu Xu, Jiaqi Ling, Lei Xiao, Jingzhou Xu, Ruike Zhang, Hao Wang, Tong Su

**Affiliations:** 1 School of Health Science and Engineering, University of Shanghai for Science and Technology, Shanghai, China; 2 Department of Medical Psychology, College of Psychology, Naval Medical University, Shanghai, China

**Keywords:** insomnia, mental health, network analysis, resilience, social support

## Abstract

**Background:**

During the COVID-19 pandemic, population mental health and sleep problems have become increasingly prominent. Social support, resilience, and quality of life are key determinants of sleep quality; however, their complex interrelationships remain insufficiently understood. This study used network analysis to examine the connections among social support, resilience, quality of life, and insomnia symptoms.

**Methods:**

We recruited 662 participants between April and May 2022. Participants completed the Social Support Rating Scale (SSRS), the Connor–Davidson Resilience Scale (CD-RISC), the World Health Organization Quality of Life—Brief (WHOQOL-BREF), and the Athens Insomnia Scale (AIS). We constructed network models with the dimensions of each scale and individual insomnia symptoms as nodes and computed centrality metrics (strength, expected influence) and bridge expected influence (BEI).

**Results:**

The network showed negative associations between insomnia symptoms and social support, resilience, and quality of life. Q2 (psychological) and R2 (strength) had the highest strength and high expected influence. R1 (tenacity), Q2 (psychological), and S1 (objective support) demonstrated the greatest positive bridge expected influence in the network, whereas I6 (sense of well-being during the day) had the greatest negative bridge expected influence within the insomnia symptom community.

**Conclusion:**

This study illuminates the complex interconnections among social support, resilience, quality of life, and insomnia symptoms in the context of prolonged pandemic control measures and identifies core and bridge nodes through network analysis. These findings provide a theoretical foundation for improving population mental health and sleep quality. Clinicians should prioritize interventions that target core nodes and bridge symptoms to enhance effectiveness.

## 1 Introduction

Quality of life (QOL) is an important concept in health and medicine. The World Health Organization (WHO) defines quality of life as “an individual’s perception of their position in the in the life in the context of the culture in which they live and in relation to their goals, expectations, standards and concerns” (1). The COVID-19 pandemic, together with the associated lockdown measures, economic disruption, and widespread fear of the disease, has profoundly affected the daily lives of people worldwide, contributing to an increased prevalence of psychological problems such as depression, anxiety, and post-traumatic stress disorder (2, 3). These psychological problems are closely associated with sleep disturbances, making sleep an important public health concern (4–7).

Sleep is a fundamental physiological process essential for maintaining physical health and overall quality of life (8). Multiple studies conducted during the pandemic showed that insomnia symptoms became increasingly common as the pandemic progressed (9–12). Insomnia is often accompanied by impaired daytime functioning, psychological problems, and varying degrees of reduced quality of life (13). From a public health perspective, sleep health should not be viewed merely as an individual nighttime issue, but as a broader determinant of physical, psychological, and social well-being. From a public health perspective, sleep health should therefore not be viewed merely as an individual concern, but rather as a multifaceted issue shaped by social and environmental conditions and closely linked to physiological, psychological, and social well-being (14).

Among the psychosocial factors associated with sleep and mental health, social support and resilience have been identified as two key protective resources. Social support refers to various forms of help and care an individual receives from others, including emotional support, instrumental support, informational support, and appraisal support (15). According to the stress-buffering hypothesis (16), social support can enhance individuals’ ability to cope with stress and protect them from the adverse effects of stressful events. Previous studies have shown that higher levels of social support are associated with fewer insomnia symptoms (17, 18), lower levels of anxiety and depression (19), and better quality of life (20).

Resilience refers to an individual’s ability to adapt positively and recover mental health when faced with adversity, trauma, tragedy, threats, or other significant stressors (21, 22). Contemporary theories emphasize that resilience is a dynamic, multisystem process that depends not only on internal personal characteristics but also on external cultural and social resources (23). Previous studies have shown that individuals with higher resilience are less likely to develop depression and anxiety symptoms under stress (24). Research conducted during the COVID-19 pandemic has further demonstrated positive associations among psychological resilience, insomnia, and quality of life (13, 25).

From the perspective of the biopsychosocial model (26) and broader public health frameworks, insomnia, social support, psychological resilience, and quality of life should not be viewed as isolated constructs. Rather, they are interrelated components of an individual’s psychosocial adaptation system. For example, social support may indirectly improve sleep and mental health by enhancing resilience (27, 28), whereas insomnia may in turn undermine perceived resilience and quality of life (13). However, most existing studies have focused only on bivariate associations or simple mediation models involving two or three of these variables (29, 30). Research simultaneously examining the complex interrelationships among all four core constructs within an integrated network framework remains limited (20, 31, 32).

Network analysis offers a useful approach for understanding such complex multivariable systems. Network analysis differs from traditional correlation or regression analyses based on total scores in that it conceptualizes variables as “nodes” within a network and directly estimates the conditional dependencies among them, typically through partial correlations, thereby enabling the visualization and quantification of the overall system structure (33, 34).

Based on the above background, this study aimed to: (1) construct and visualize a network of associations linking dimensions of social support, resilience, and quality of life with specific insomnia symptoms; and (2) identify the central and bridge nodes within this network to explain the key pathways connecting these constructs. The data were collected between April and May 2022, when China was still enforcing its dynamic zero-COVID strategy, thus capturing the psychosocial status of the general population under prolonged pandemic control measures. The findings of this study may provide a more precise theoretical foundation for developing interventions to improve sleep and quality of life by strengthening social support and psychological resilience in the context of major public health emergencies.

## 2 Materials and methods

### 2.1 Study design and participants

This study employed a cross-sectional survey design. From April to May 2022, a total of 662 participants were recruited through voluntary participation in an online survey, primarily in Shanghai, China. Participants represented diverse occupations, age, and educational backgrounds, including university students, community workers, and medical personnel. After excluding 36 invalid questionnaires (defined as those with incorrect, incomplete, or repetitive responses for multiple consecutive questions), a final sample of 626 valid questionnaires was collected, yielding a valid response rate of 94.56%. Eligible participants were those who voluntarily agreed to participate and were able to read, understand, and complete the questionnaire independently. Individuals with a history of severe psychiatric disorders or those unable to complete the questionnaire on their own were excluded. All participants completed the online survey voluntarily after providing informed consent. This study received approval from the Ethics Committee of Naval Medical University.

### 2.2 Measures

The survey was conducted on Wenjuanxing^1^, a widely used online survey platform in China. The study utilized four self-report instruments: the Social Support Rating Scale (SSRS), the Connor-Davidson Resilience Scale (CD-RISC), the World Health Organization Quality of Life-BREF (WHOQOL-BREF), and the Athens Insomnia Scale (AIS). A self-designed general information questionnaire collected demographic characteristics, including age, gender, education level, and occupation.

#### 2.2.1 Social support rating scale

The Social Support Rating Scale (SSRS) was used to measure social support. This 10-item scale, scored on a 4-point Likert scale (1–4), comprises three measurable dimensions: objective support (3 items), subjective support (4 items), and utilization of support (3 items) (35). Objective support refers to tangible assistance, subjective support relates to an individual’s perceived support, and utilization of support reflects how individuals engage with available support. Higher total scores indicate greater social support.

#### 2.2.2 Chinese version of the Connor–Davidson resilience scale

The Connor-Davidson Resilience Scale (CD-RISC) was employed to assess psychological resilience. This 25-item scale, scored on a 5-point Likert scale (0–4), includes three measurable dimensions: tenacity (13 items), strength (8 items), and optimism (4 items) (36). Higher scores reflect stronger adaptive capacity (37).

#### 2.2.3 World Health Organization quality of life-BREF

The World Health Organization Quality of Life-BREF (WHOQOL-BREF) is a shortened version of the WHOQOL-100. Extensive research has demonstrated that the WHOQOL-BREF can effectively substitute the WHOQOL-100 in measuring scores across various domains related to QOL. This 26-item scale generates scores for four domains: physical (7 items), psychological (6 items), social relationships (3 items), and environment (8 items). It also includes two independent questions on overall health and overall quality of life for practical application. The results are comparable to those of the WHOQOL-100. Scores range from 0 to 100, with higher scores indicating better quality of life (38).

#### 2.2.4 Athens insomnia scale

The Athens Insomnia Scale (AIS) is a self-reported psychometric tool designed to quantify sleep difficulties according to ICD-10 criteria. It consists of 8 items: the first five items pertain to sleep induction, nocturnal awakenings, final awakenings, total sleep duration, and sleep quality; the latter three items address daytime well-being, functional capacity, and sleepiness (39). Scores range from 0 to 24, with higher AIS scores indicating a greater level of insomnia.

### 2.3 Data analysis

Descriptive statistics were performed on the sociodemographic data using SPSS 27.0, and Spearman’s correlation analysis was conducted for the four variables. Network analysis was performed using R (Version 4.5.0) and RStudio (Version 2024.12.1 + 563). A social support-resilience-quality of life-insomnia symptoms network was constructed and visualized using the R package qgraph (version 1.9.8) (40), solid gray lines represent positive correlations, while dashed red lines represent negative correlations. Nodes in the network were categorized into four communities based on their origin: social support, resilience, quality of life, and insomnia symptoms. Before network analysis, the included data underwent Z-transformation to ensure consistency. A Gaussian Graphical Model (GGM) was used to estimate the network. To obtain a sparse network, a combination of the least absolute shrinkage and selection operator (LASSO) and the extended Bayesian Information Criterion (EBIC) was applied for network construction (41). LASSO shrinks small partial correlation coefficients toward zero, resulting in a sparse and interpretable network. EBIC was used to select the optimal model by balancing goodness of fit and model complexity. Following previous recommendations, the EBIC hyperparameter γ was set to 0.5 (41). This procedure improves the accuracy of network estimation and reduces the likelihood of including spurious edges due to random noise (42).

For centrality indices, we calculate each node’s strength centrality (41, 43) and expected influence (EI). Bridge expected influence (BEI) was computed using the *networktools* package (version 1.6). Previous research has suggested that betweenness and closeness centrality are less suitable for psychological networks (43, 44), despite their effectiveness in other network contexts (45); therefore, the present study focuses on reporting strength centrality. Strength centrality is defined as the sum of the absolute weights of edges connecting a node to all other nodes. Higher strength centrality indicates a stronger direct influence within the network. EI is similar to strength centrality but retains the positive or negative sign of the edges, reflecting the node’s overall influence (46). BEI quantifies a node’s ability and strength to connect different communities within the network, defined as the sum of edge weights between that node and all nodes in other communities (45).

To evaluate the accuracy of the network, we conducted a bootstrap difference test using the *bootnet* package (version 1.6). This test allowed us to assess the precision of the estimated network metrics. We used a non-parametric bootstrapping method (1,000 bootstrap samples, *α* = 0.05) to calculate the 95% confidence intervals (CIs) of edge weight values to assess accuracy (47). For stability, a case-dropping bootstrapping method was used to calculate the correlation stability (CS) coefficients for strength centrality, EI, and BEI. A CS coefficient of at least 0.25 is acceptable, with values greater than 0.5 considered optimal (41).

## 3 Results

### 3.1 Participants’ demographic information

This study included 626 participants. The sample comprised 453 females (72.4%) and 173 males (27.6%), with the majority aged 18–25 years (75.7%). Regarding education, 91 participants (14.5%) held a junior college degree or lower, while 535 (85.5%) had a bachelor’s degree or higher. Students constituted 443 (70.8%) of the sample. Sociodemographic characteristics are detailed in Supplementary Table S1. Total scores and factor scores of the four scales are presented in Table 1.

**Table 1:** Scale scores of all participants.

Variable	Abbreviation	Mean	SD
Social Support Rating Scale (SSRS) Total score		37.45	6.70
Objective support	S1	6.80	2.37
Subjective support	S2	21.99	4.80
Support availability	S3	8.59	1.77
Connor–Davidson Resilience Scale (CD-RISC) Total score		87.42	14.10
Tenacity	R1	44.62	8.23
Strength	R2	29.30	4.77
Optimism	R3	13.5	2.57
World Health Organization Quality of Life—Brief (WHOQOL-BREF)			
Physical	Q1	61.24	14.37
Psychological	Q2	60.17	16.67
Social relationship	Q3	61.13	18.16
Environment	Q4	60.45	16.60
Athens Insomnia Scale (AIS) Total score		5.64	3.67
Sleep induction	I1	1.93	0.78
Awakenings during the night	I2	1.72	0.75
Early awakening	I3	1.67	0.73
Sleep duration	I4	1.70	0.71
Quality of sleep	I5	1.70	0.72
Sense of well-being during the day	I6	1.42	0.63
Functioning during the day	I7	1.61	0.69
Sleepiness during the day	I8	1.86	0.64

### 3.2 Correlation analysis

**Figure 1:**
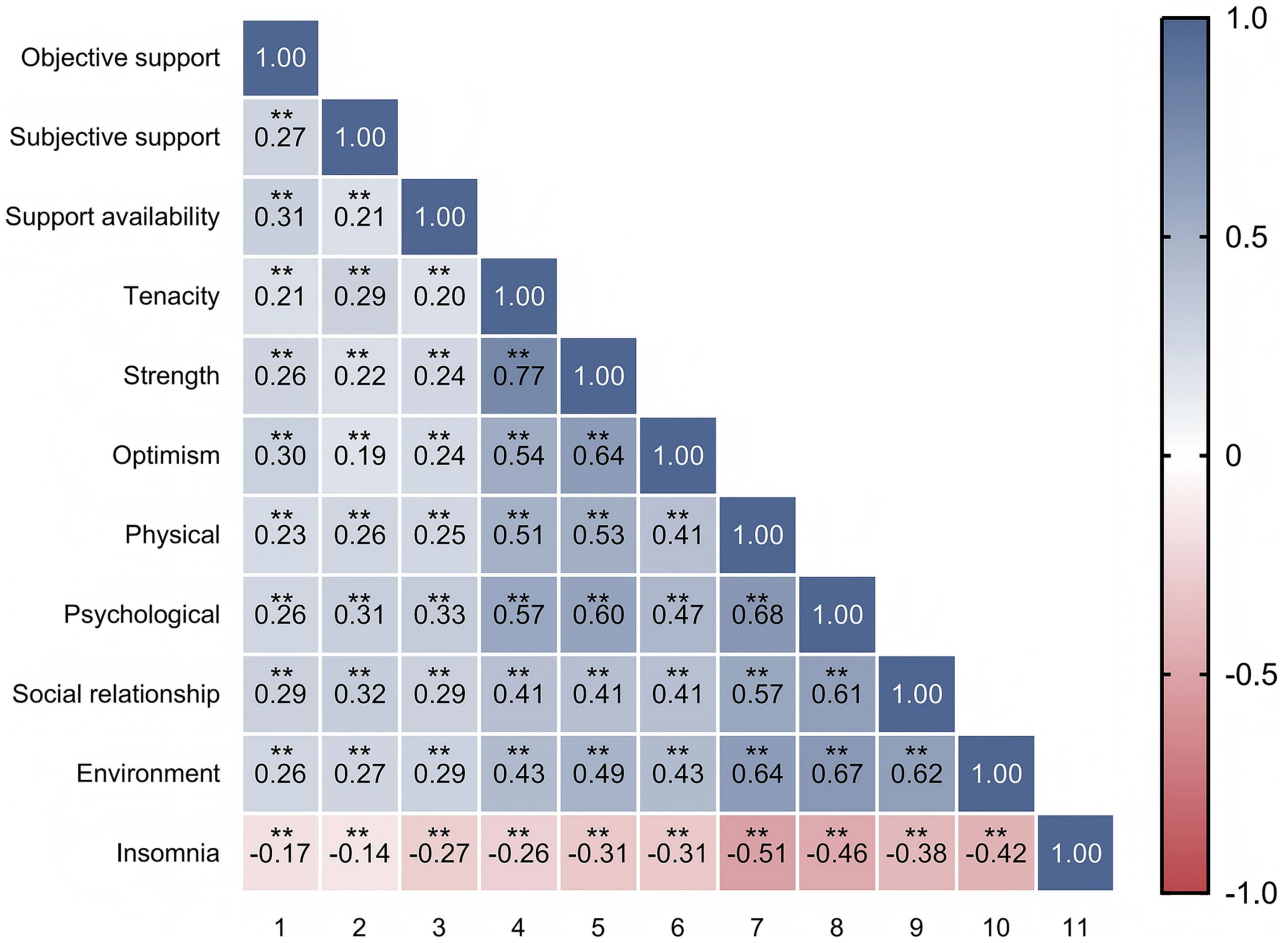
Spearman’s bivariate correlation matrix of variables. *p < 0.05, **p < 0.01.

Bivariate Spearman’s correlation analysis revealed significant positive correlations among the three dimensions of social support, the three dimensions of resilience, and the four domains of quality of life. Conversely, insomnia showed significant negative correlations with all dimensions of social support, resilience, and quality of life (Figure 1).

### 3.3 Social support-resilience-quality of life-insomnia symptoms network

**Figure 2:**
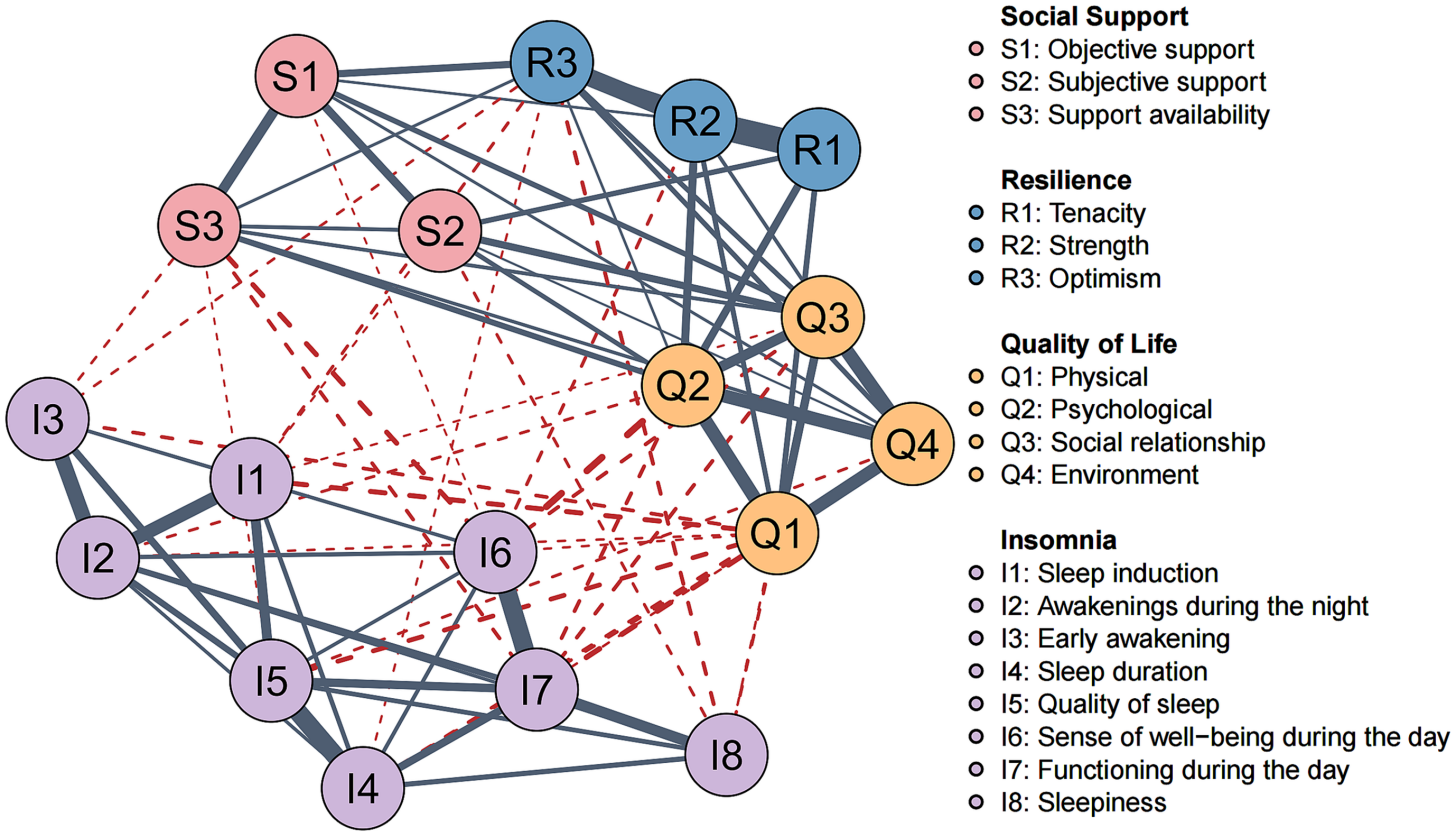
Social support-resilience-quality of life-insomnia symptoms network. Solid blue-gray lines represent positive correlations, dashed red lines represent negative correlations, and thicker edges indicate stronger correlations.

The social support – resilience – quality of life – insomnia symptoms network, comprising 18 variables, is illustrated in Figure 2. An initial estimation yielded 153 edges, which, after regularization, resulted in 83 non-zero edges in the entire network. Notably, the three components of social support and the three components of resilience exhibited significant positive correlations, with S1 (Objective Support) and R3 (Optimism) showing a strong association (weight = 0.11). The four domains of quality of life (Q1–Q4) were tightly interconnected, with Q1 (Physical) and Q2 (Psychological) positively correlated (weight = 0.24), and Q3 (Social Relationships) and Q4 (Environment) also positively correlated (weight = 0.25). Within the insomnia symptom cluster, strong positive correlations were observed between I4 (Sleep Duration) and I5 (Quality of Sleep), and between I6 (Sense of Well-being During the Day) and I7 (Functioning During the Day) (weights = 0.36, 0.36, respectively). All individual insomnia symptoms (I1-I7) were negatively correlated with the social support, resilience, and quality of life communities. Specifically, I6 (Sense of Well-being During the Day) showed strong negative correlations with S3 (Support availability) and Q2 (Psychological) (weights = −0.07, −0.10, respectively), while I8 (Sleepiness During the Day) was strongly negatively correlated with R3 (Optimism) (weight = −0.04). Supplementary Table S2 provides all edge weights in the network. As shown in Supplementary Figure S1, the 95% confidence intervals of the edge weights indicate acceptable network edge weight accuracy. The differential test for edge weights is presented in Supplementary Figure S2.

### 3.4 Centrality results

**Figure 3:**
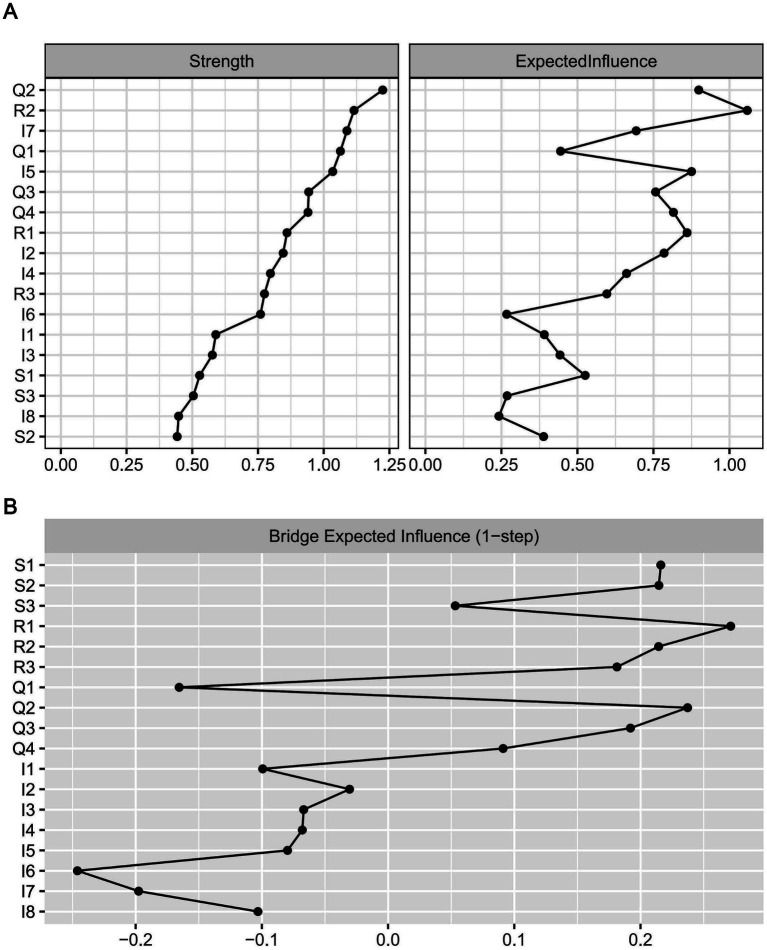
Centrality results and bridge expected influence in the social support-resilience-quality of life-insomnia network. **(A)** Raw scores for strength centrality and expected influence in the social support-resilience-quality of life-insomnia network. **(B)** Raw scores for bridge expected influence in the social support-resilience-quality of life-insomnia network.

The estimated strength, expected influence (EI), and bridge expected influence (BEI) of the network are presented in Figure 3. As shown in Figure 3A, Q2 (Psychological) (Rs = 1.22) and R2 (Strength) (Rs = 1.12) exhibited the highest strength values, indicates that these nodes possess the strongest connectivity with other nodes within the network. Q2 (Psychological) (EI = 0.90) and R2 (Strength) (EI = 1.06) also demonstrated high EI values, indicating their strong association with neighboring nodes. Bootstrap difference tests for strength centrality and expected influence are provided in Supplementary Figures S3, S4. Based on the correlation stability coefficients, both strength (CS = 0.751) and expected influence (CS = 0.751) were deemed stable (Supplementary Figure S5). Figure 3B illustrates the BEI values for each node in the network. R1, Q2, and S1 showed the largest positive BEI values within the resilience, quality of life, and social support communities, respectively (BEI = 0.27, 0.24, 0.22). Nodes with higher positive BEI values are considered to exert a positive influence in connecting different communities within the network. Conversely, I6 exhibited the largest negative BEI value within the insomnia symptoms community (BEI = −0.25). The results of the differential test for BEI are provided in Supplementary Figure S6. The stability coefficient for BEI was 0.751, indicating good stability (Supplementary Figure S7).

## 4 Discussion

To our knowledge, this study is the first to use network analysis to examine the psychosocial system involving social support, resilience, quality of life, and insomnia symptoms in the context of prolonged pandemic control measures. The resulting network revealed complex associations among these variables and identified potentially important central and bridge nodes. The findings are discussed below in relation to the network structure.

First, the network structure confirmed a general pattern of negative associations between protective factors and insomnia symptoms. Specifically, all dimensions of social support, resilience, and quality of life were negatively connected with insomnia symptom nodes. This finding is consistent with a large body of cross-sectional research showing that higher levels of social support, stronger resilience, and better quality of life are generally associated with fewer reported insomnia symptoms (19, 48, 49). Particularly noteworthy were several strong negative edges, including those between R3“optimism” and I8“sleepiness during the day,” as well as between S3“support availability,” Q2“psychological,” and I6“sense of well-being during the day.” These findings suggest that individuals who perceive lower availability of social support or report poorer psychological health may be more likely to experience pronounced daytime impairment related to insomnia, such as sleepiness and low mood (50, 51). According to the stress-buffering hypothesis (16) and prior evidence (52), social support and positive psychological characteristics, such as optimism, may be associated with lower levels of psychological distress and physiological arousal under stress by providing coping resources or altering stress appraisal, both of which are common contributors to insomnia (53, 54). However, the cross-sectional nature of the data precludes any inference regarding directionality. These broadly negative associations may also reflect a reverse pathway, whereby daytime fatigue and emotional distress resulting from insomnia diminish individuals’ ability to perceive and utilize social support and lower their evaluations of their own psychological state and optimism, thereby contributing to a mutually reinforcing cycle.

Second, an in-depth analysis of the patterns of connection across different dimensions of social support revealed the importance of support matching and support perception. The strongest positive correlations were observed between S1“objective support” and R3“optimism” within social support and resilience, and between S2“subjective support” and Q3“social relationship” within social support and quality of life. This pattern is consistent with the matching hypothesis proposed by Cohen and Wills (16), which suggests that social support exerts its strongest buffering effect when the form of support provided corresponds to the specific demands imposed by the stressor. The positive correlation between S1 and R3 is consistent with recent studies (55, 56). In the context of this study, the strong association between objective support and optimism may suggest that, during the uncertain period of pandemic control, tangible material or instrumental resources are crucial for maintaining individuals’ positive future-oriented mindset. Emotional subjective support, on the other hand, directly forms the core experience of satisfaction with social relationships. Good social relationships can provide individuals with emotional, informational, and instrumental support, and these objectively existing relationships form the basis for an individual’s perception of support (16, 57).

Third, centrality analysis indicated that Q2 “psychological” and R2 “strength” were among the most central nodes in the network, suggesting that they occupied central positions within the overall association structure. Q2 from quality of life and R2 from resilience showed a positive correlation and also exhibited high strength centrality and high Expected Influence (EI) values across the entire network. This suggests that Q2 and R2 may play especially influential roles in the overall psychosocial system, with the potential for their effects to extend to multiple other nodes. This finding suggests that psychological health and strength may represent nodes of relatively high systemic importance within the psychosocial network formed by these variables. Specifically, psychological health may be broadly associated with individuals’ evaluations of other life domains, such as social relationships and the environment, as well as with perceived social support and experience of insomnia symptoms (58, 59). Similarly, changes in resilience may also have a broad impact on their emotional state, life satisfaction, and sleep responses under stress (36). This result is consistent with the biopsychosocial model (26) and the systemic perspective on resilience (23), both of which emphasize that mental health and resilience are not isolated attributes but are embedded within an individual’s broader psychosocial adaptation network. From an intervention perspective, psychological health and resilience may therefore represent promising targets for priority attention, as improvements in these domains may, in theory, be associated with broader beneficial changes across the overall system (21, 60).

Fourth, bridge centrality analysis further identified key nodes linking different communities within the network. The results showed that R1“tenacity” had the largest positive BEI in the entire network, indicating that it serves as an active bridge connecting the community of external protective resources, such as social support, with the community of internal protective resources, such as resilience and quality of life. This finding is consistent with previous research demonstrating that highly resilient individuals are more likely to adopt proactive, problem-focused coping strategies (61), which in turn positively influence their perceived quality of life. In contrast, I6 “sense of well-being during the day” had the largest negative BEI, indicating that it had the strongest overall negative cross-community associations. This suggests that impaired daytime well-being may not only be a consequence of insomnia, but may also in turn substantially hinder individuals’ ability to access and utilize social support and to maintain favorable psychological states (49), thereby contributing to the emergence and maintenance of a mutually reinforcing maladaptive cycle. This finding highlights the central role of daytime functioning within the overall network and is consistent with the rationale of cognitive behavioral therapy for insomnia (CBT-I), which emphasizes the restoration of daytime functioning. It further suggests that, in addition to addressing insomnia symptoms, interventions should place particular emphasis on improving daytime mood and reducing daytime impairment, as this may more effectively interrupt this cycle and yield broader overall benefits (62).

This study offers new directions and insights for interventions targeting insomnia and mental health. Beyond traditional pharmacological and first-line cognitive behavioral therapies for insomnia, interventions could focus on enhancing resilience, particularly by fostering growth-oriented psychological traits in adversity and leveraging social support. This approach could systematically improve individuals’ overall adaptive capacity and mental health. However, this study has certain limitations. First, the demographic information collected was relatively limited, including only gender, age, education, and occupation, which may have restricted the examination of other potentially important influencing factors. Second, the sample size was relatively small and was composed mainly of young adults and students, which may limit the generalizability of the findings. Third, all variables were assessed using self-report measures, which may have introduced common method bias. In addition, potentially relevant behavioral variables, such as physical activity, sedentary behavior, sleep hygiene, and screen exposure time, were not included, limiting the interpretation of factors related to insomnia. Future studies may consider incorporating these variables into the network to provide a more comprehensive understanding of the factors related to insomnia. Finally, the cross-sectional design of this study precluded any assessment of temporal or causal relationships and did not allow the dynamic changes in these variables over time to be captured. Future studies incorporating longitudinal data into network models may provide a more comprehensive understanding of the psychosocial health system.

## 5 Conclusion

In summary, this study, utilizing network analysis, for the first time demonstrated the complex interplay between insomnia and multiple factors in the context of prolonged pandemic control measures. Insomnia is not an isolated nocturnal physiological issue but is deeply intertwined with an individual’s daytime functioning, resilience, and social support system. In responding to major public health crises, maintaining individuals’ daytime mood and enhancing positive psychological resources offer new intervention strategies for preventing and alleviating sleep disorders and broader mental health issues.

## Data Availability

The raw data supporting the conclusions of this article will be made available by the authors, without undue reservation.

## Footnotes

1
https://www.wjx.cn
