# Supplementary material for: Network analysis of social support, resilience, quality of life, and insomnia under pandemic control measures
Source: Front Public Health. 2026 May 14;14:1801742. doi: 10.3389/fpubh.2026.1801742 (PMC13215862; doi:10.3389/fpubh.2026.1801742)
Supplement: Supplementary file 1 [file Table_1.docx]

Supplementary Material

Table S1 Demographic Information

| Characteristic | n (%) | Resilience | | Social support | | Physical | | Psychological | | Social relationship | | Environment | | Insomnia | |
| --- | --- | --- | --- | --- | --- | --- | --- | --- | --- | --- | --- | --- | --- | --- | --- |
|  |  | Z/H | P | Z/H | P | Z/H | P | Z/H | P | Z/H | P | Z/H | P | Z/H | P |
| Gender |  | -2.578 | 0.01 | -1.141 | 0.254 | -1.270 | 0.204 | -0.417 | 0.676 | -1.518 | 0.129 | -0.518 | 0.604 | -1.548 | 0.122 |
| Male | 173 (27.6%) |  |  |  |  |  |  |  |  |  |  |  |  |  |  |
| Female | 453 (72.4%) |  |  |  |  |  |  |  |  |  |  |  |  |  |  |
| Age |  | 20.883 | ＜0.001 | 23.262 |  | 17.115 | 0.004 | 8.540 | 0.129 | 12.362 | 0.03 | 7.779 | 0.169 | 6.754 | 0.240 |
| < 18 | 11 (1.8%) |  |  |  |  |  |  |  |  |  |  |  |  |  |  |
| 18-25 | 474 (75.7%) |  |  |  |  |  |  |  |  |  |  |  |  |  |  |
| 26-30 | 36 (5.8%) |  |  |  |  |  |  |  |  |  |  |  |  |  |  |
| 31-40 | 43 (6.9%) |  |  |  |  |  |  |  |  |  |  |  |  |  |  |
| 41-50 | 44 (7.0%) |  |  |  |  |  |  |  |  |  |  |  |  |  |  |
| ≥ 51 | 18 (2.9%) |  |  |  |  |  |  |  |  |  |  |  |  |  |  |
| Education |  | 20.770 | 0.001 | 5.867 | 0.118 | 14.559 | 0.002 | 7.427 | 0.059 | 8.592 | 0.035 | 2.336 | 0.506 | 6.252 | 0.10 |
| High school or below | 30 (4.8%) |  |  |  |  |  |  |  |  |  |  |  |  |  |  |
| Junior college | 61 (9.7%) |  |  |  |  |  |  |  |  |  |  |  |  |  |  |
| Bachelor's degree | 482 (77.0%) |  |  |  |  |  |  |  |  |  |  |  |  |  |  |
| Master's degree or above | 53 (8.5%) |  |  |  |  |  |  |  |  |  |  |  |  |  |  |
| Occupation |  | 10.633 | 0.031 | 11.645 | 0.02 | 7.805 | 0.099 | 5.110 | 0.276 | 11.211 | 0.024 | 0.825 | 0.935 | 4.820 | 0.306 |
| Student | 443 (70.8%) |  |  |  |  |  |  |  |  |  |  |  |  |  |  |
| Company employee | 81 (12.9%) |  |  |  |  |  |  |  |  |  |  |  |  |  |  |
| Civil servant | 50 (8.0%) |  |  |  |  |  |  |  |  |  |  |  |  |  |  |
| Self-employed | 14 (2.2%) |  |  |  |  |  |  |  |  |  |  |  |  |  |  |
| Other | 38 (6.1%) |  |  |  |  |  |  |  |  |  |  |  |  |  |  |

Table S2 All the edge weights in the Social Support-Resilience-Quality of Life-Insomnia Network

|  | S1 | S2 | S3 | R1 | R2 | R3 | Q1 | Q2 | Q3 | Q4 | I1 | I2 | I3 | I4 | I5 | I6 | I7 | I8 |
| --- | --- | --- | --- | --- | --- | --- | --- | --- | --- | --- | --- | --- | --- | --- | --- | --- | --- | --- |
| S1 | 0 |  |  |  |  |  |  |  |  |  |  |  |  |  |  |  |  |  |
| S2 | .134 | 0 |  |  |  |  |  |  |  |  |  |  |  |  |  |  |  |  |
| S3 | .175 | .04 | 0 |  |  |  |  |  |  |  |  |  |  |  |  |  |  |  |
| R1 | 0 | .071 | 0 | 0 |  |  |  |  |  |  |  |  |  |  |  |  |  |  |
| R2 | .015 | 0 | 0 | .509 | 0 |  |  |  |  |  |  |  |  |  |  |  |  |  |
| R3 | .11 | 0 | .02 | .08 | .335 | 0 |  |  |  |  |  |  |  |  |  |  |  |  |
| Q1 | 0 | 0 | 0 | .079 | .065 | 0 | 0 |  |  |  |  |  |  |  |  |  |  |  |
| Q2 | 0 | .056 | .082 | .121 | .128 | .013 | .242 | 0 |  |  |  |  |  |  |  |  |  |  |
| Q3 | .073 | .11 | .038 | 0 | 0 | .064 | .136 | .178 | 0 |  |  |  |  |  |  |  |  |  |
| Q4 | .02 | .004 | .031 | 0 | .035 | .064 | .232 | .241 | .251 | 0 |  |  |  |  |  |  |  |  |
| I1 | 0 | -.005 | 0 | 0 | 0 | -.026 | -.064 | 0 | -.005 | 0 | 0 |  |  |  |  |  |  |  |
| I2 | 0 | 0 | 0 | 0 | 0 | 0 | -.01 | -.02 | 0 | 0 | .246 | 0 |  |  |  |  |  |  |
| I3 | 0 | 0 | -.015 | 0 | 0 | -.016 | -.037 | 0 | 0 | 0 | .022 | .283 | 0 |  |  |  |  |  |
| I4 | 0 | 0 | 0 | 0 | 0 | -.006 | -.031 | 0 | 0 | -.031 | .055 | .03 | .055 | 0 |  |  |  |  |
| I5 | 0 | 0 | 0 | 0 | 0 | 0 | -.058 | 0 | 0 | -.021 | .167 | .091 | .114 | .356 | 0 |  |  |  |
| I6 | -.001 | 0 | -.065 | 0 | -.029 | 0 | -.009 | -.104 | -.038 | 0 | 0 | .048 | .035 | .042 | .033 | 0 |  |  |
| I7 | 0 | 0 | -.037 | 0 | 0 | 0 | -.072 | -.039 | -.04 | -.01 | 0 | .085 | 0 | .124 | .136 | .355 | 0 |  |
| I8 | 0 | -.022 | 0 | 0 | 0 | -.042 | -.029 | 0 | -.011 | 0 | 0 | .03 | 0 | .067 | .057 | 0 | .191 | 0 |

Note:S1,objective support;S2,subjective support;S3,support availability;R1,tenacity;R2,strength;R3,Optimism;Q1,Physical;Q2,Psychological;Q3,Social relationship;Q4,Environment;I1,Sleep induction;I2,Awakenings during the night;I3,Early awakening;I4,Sleep duration;I5,Quality of sleep;I6,Sense of well-being during the day;I7,Functioning during the day;I8,Sleepiness during the day


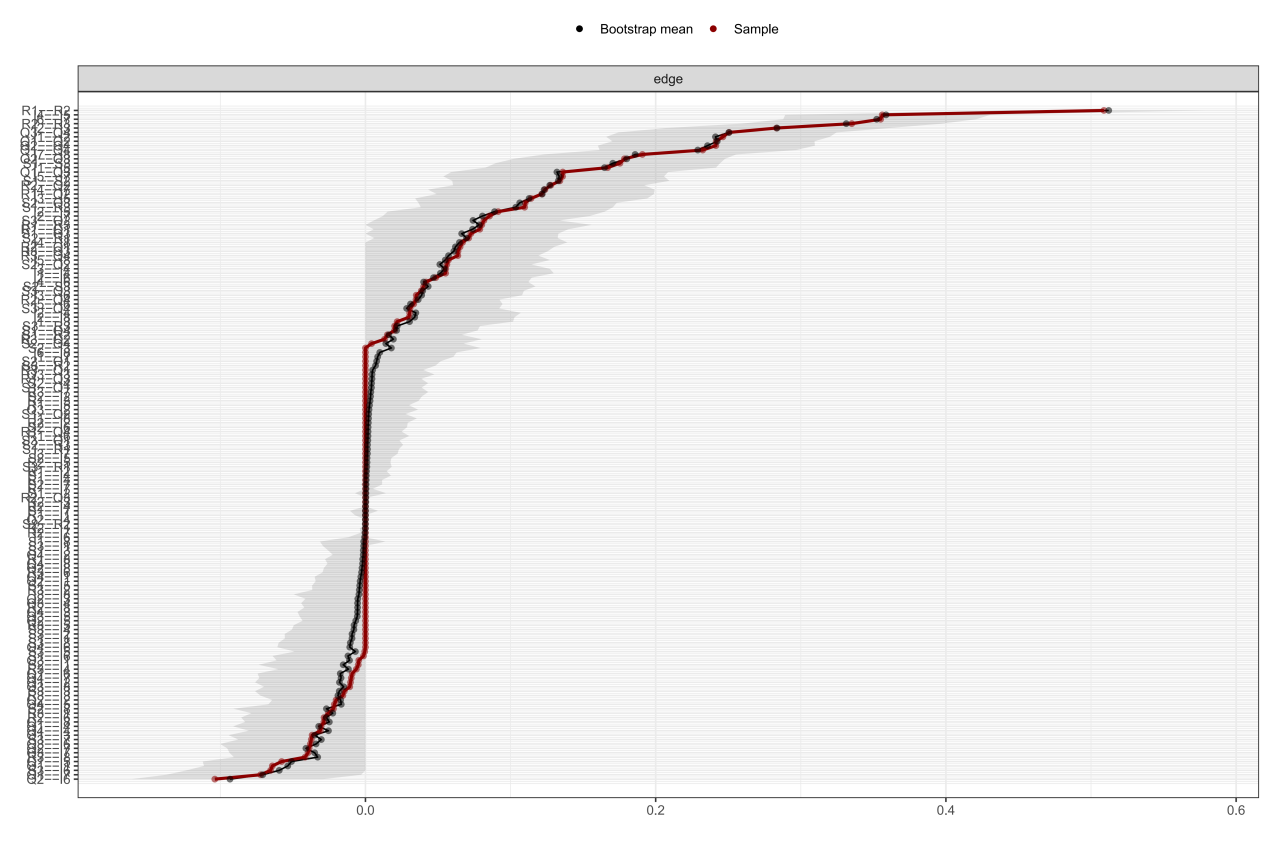


Supplementary Figure 1 Bootstrapped confidence intervals of estimated edge-weights in the Social Support-Resilience-Quality of Life-Insomnia Network

Note: The x-axis indicates the edge weights and the y-axis indicates the edges.The red line indicates the sample value and the gray area the bootstrapped CIs.


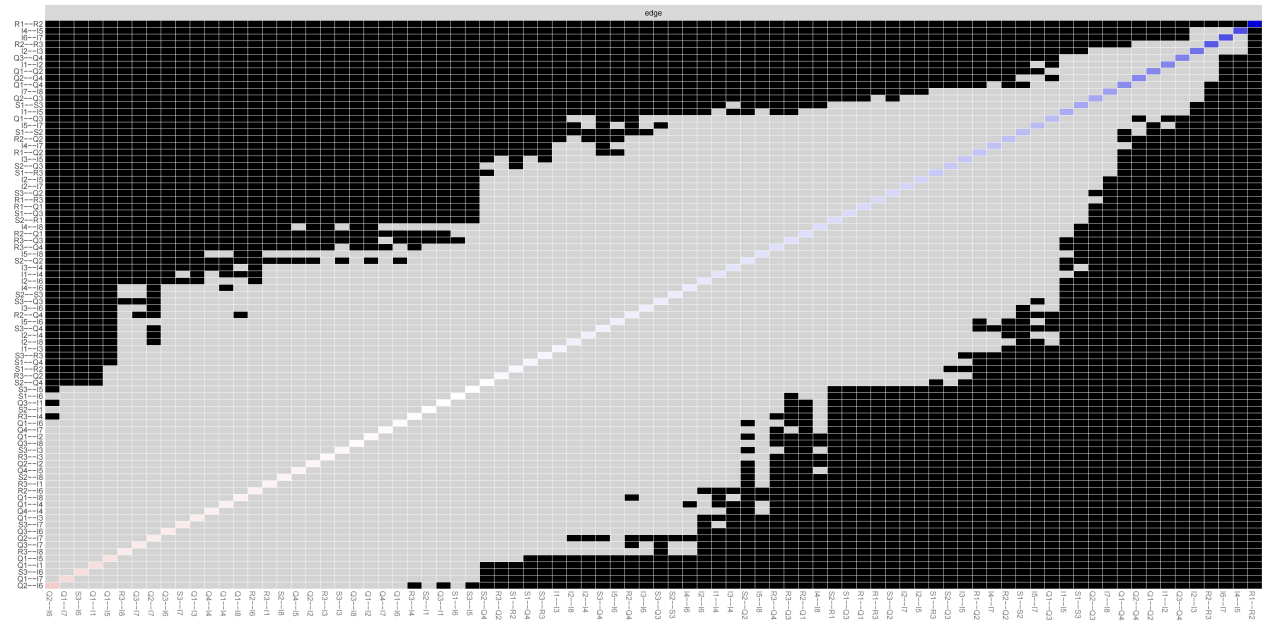


Supplementary Figure 2 Bootstrapped difference test for edge weights in the Social Support-Resilience-Quality of Life-Insomnia Network

Note:Gray boxes indicate edge weights that do not differ significantly from one another, while black boxes indicate edge weights that do differ significantly.


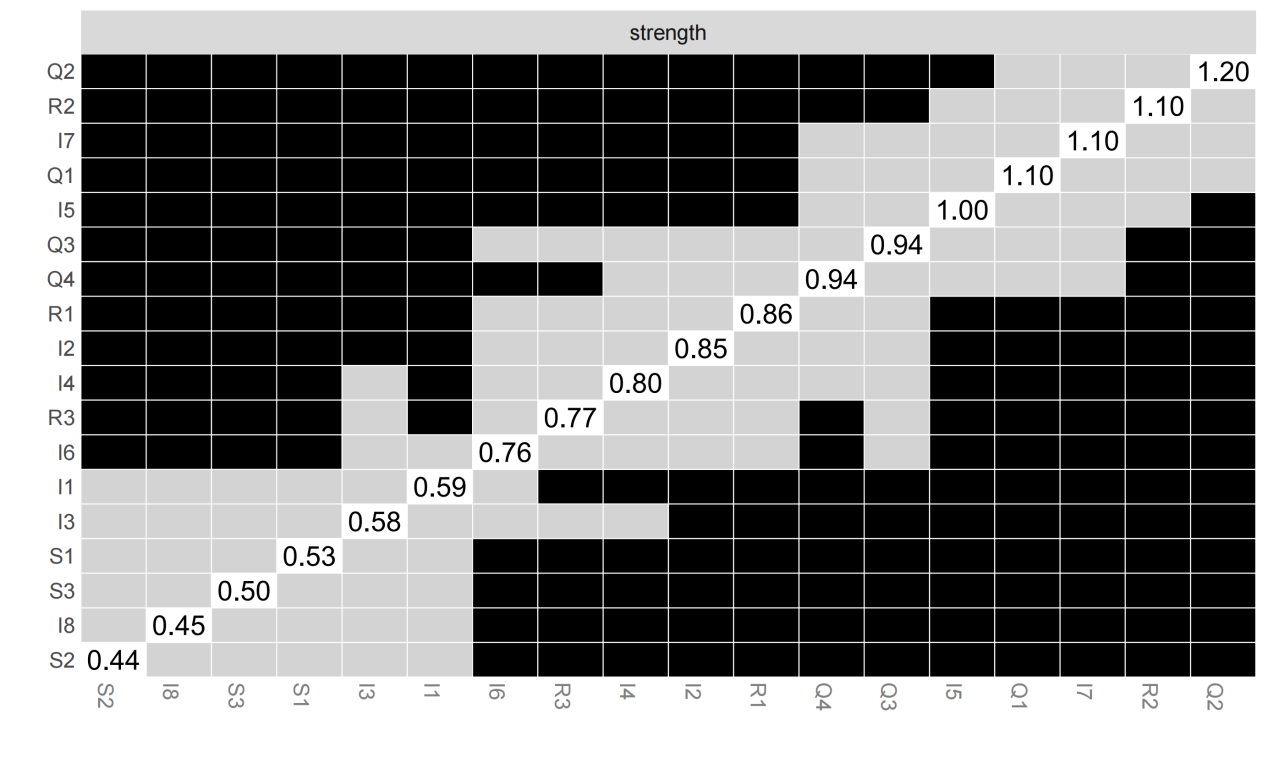


Supplementary Figure 3. Bootstrapped difference test for node strength of the Social Support-Resilience-Quality of Life-Insomnia Network

Note:Grey boxes indicate non-significant differences,while black boxes represent significant differences in strength between two variables.


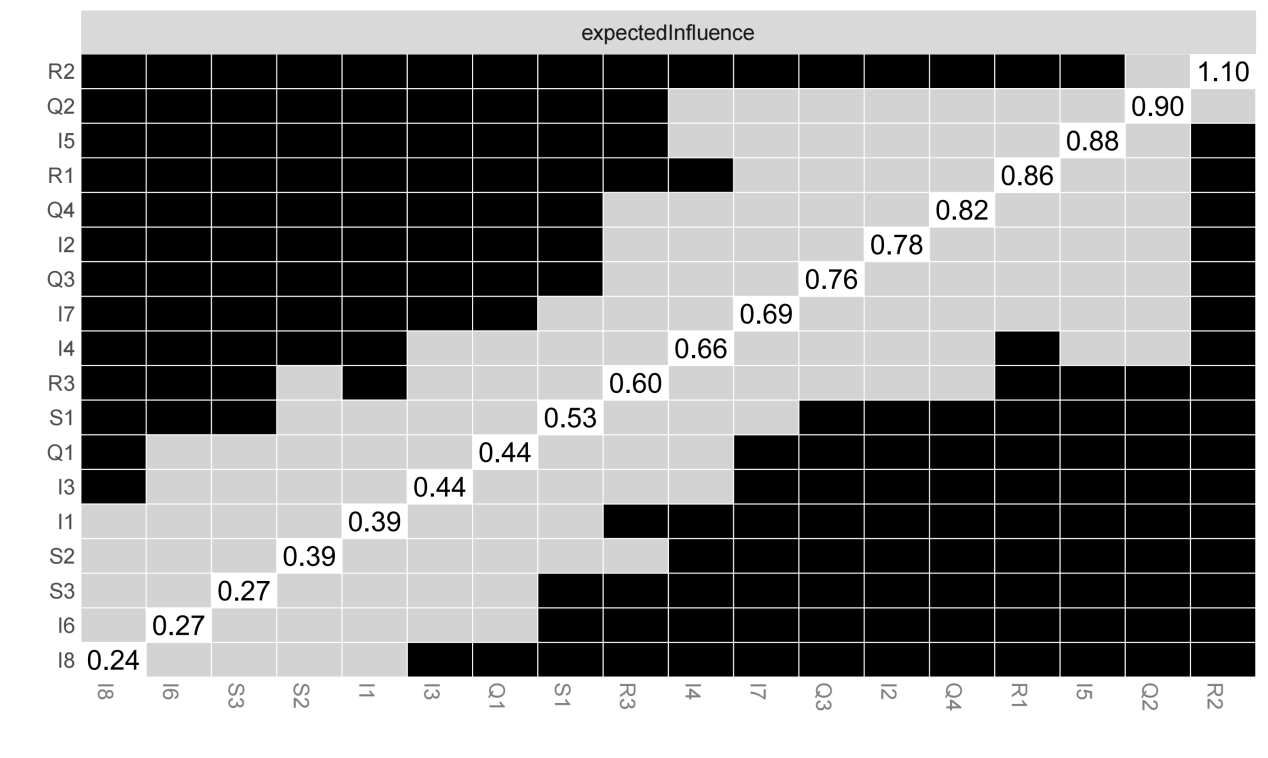


Supplementary Figure 4. Bootstrapped difference test for node expected influence of the Social Support-Resilience-Quality of Life-Insomnia Network

Note:Grey boxes indicate non-significant differences,while black boxes represent significant differences in strength between two variables.


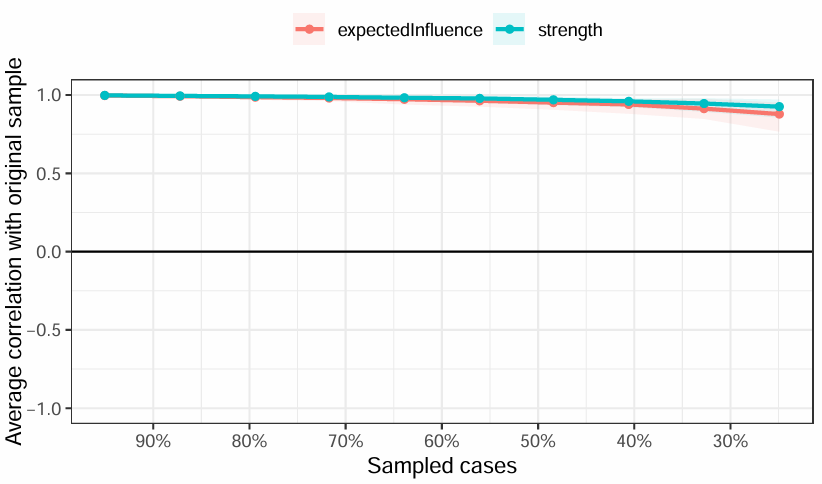


Supplementary Figure 5 Average correlations between centrality indices (strength and expected influence) of networks sampled using the drop-persons procedure and the original sample.

Note:The red line represents the mean of the expected influence of the node, and the blue line represents the mean of the node strength,areas indicate the range from the 2.5th quantile to the 97.5th quantile.


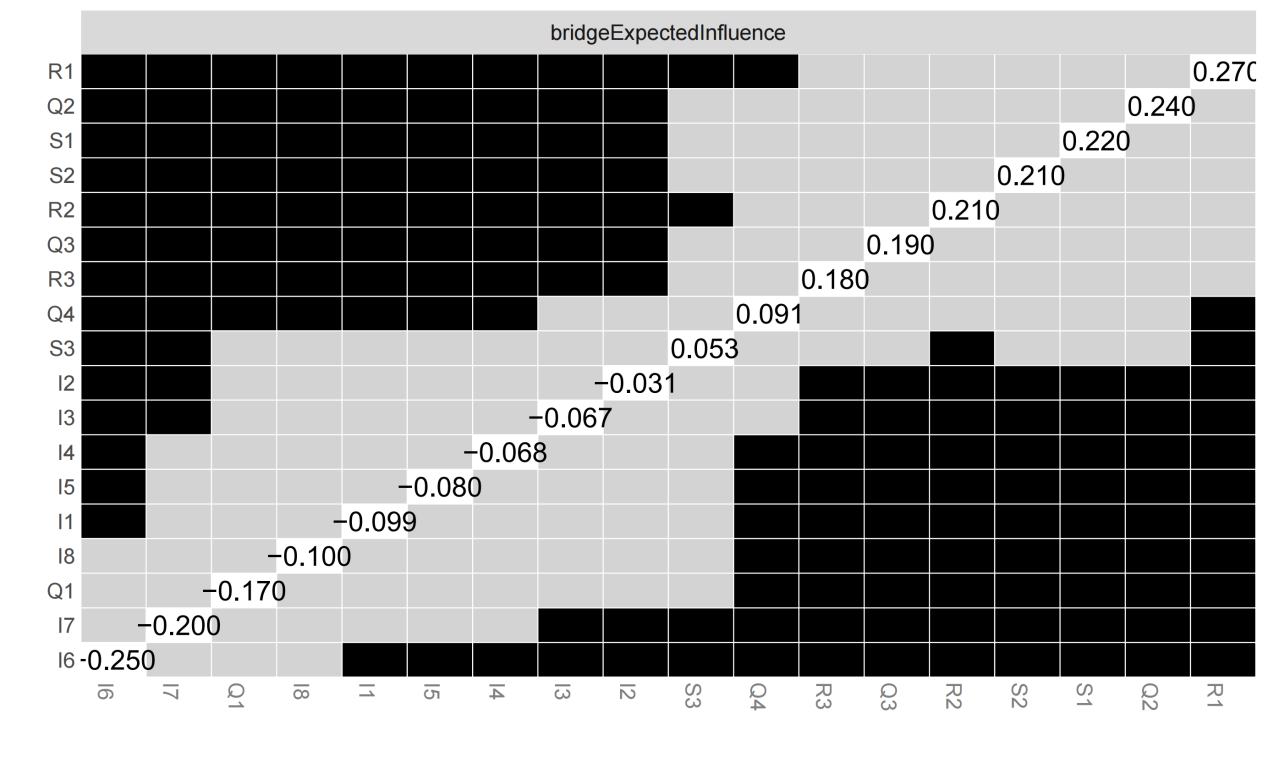


Supplementary Figure 6 Bootstrapped difference test for node bridge expected influences in the Social Support-Resilience-Quality of Life-Insomnia Network

Note: Gray boxes indicate node bridge expected influences that do not differ significantly from one another, while black boxes indicate node bridge expected influences that do differ significantly.


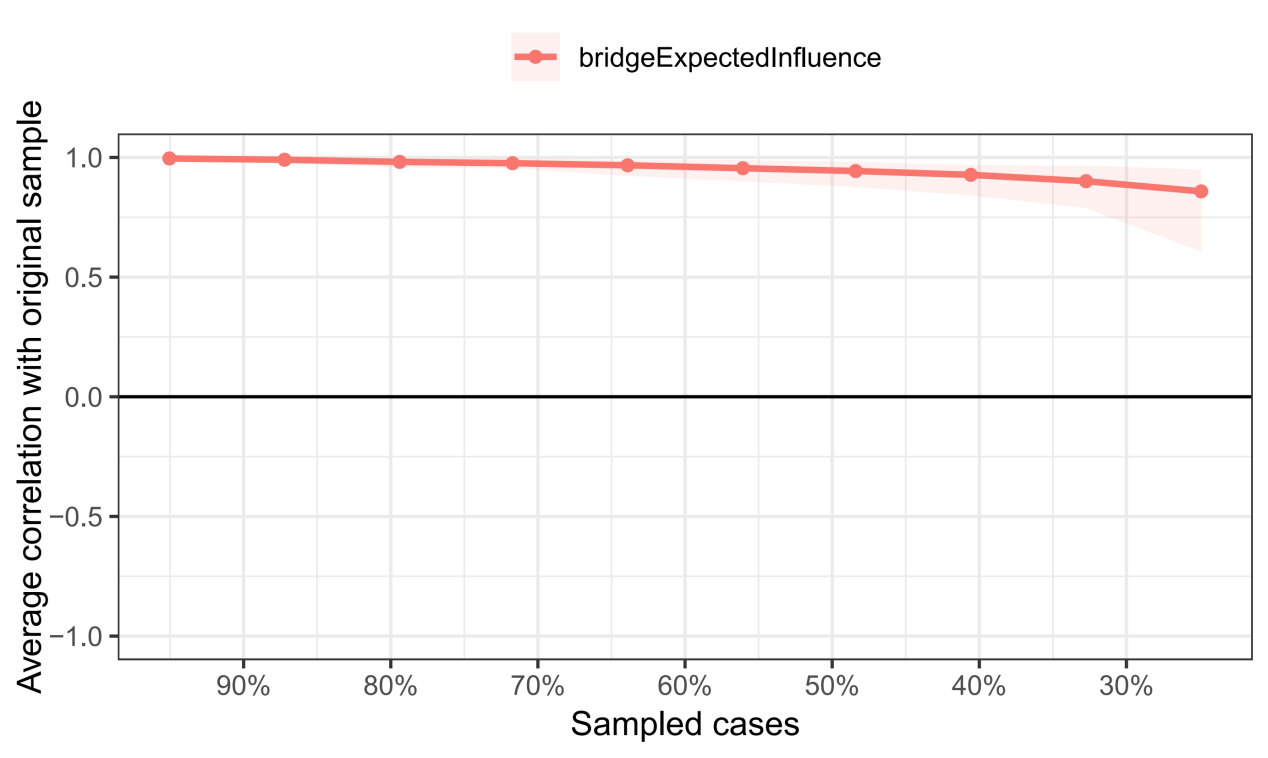


Supplementary Figure 7 The average correlation between bridge expected influence estimated from networks sampled using the drop-persons procedure and that from the original network.

Note: The red bar represents the average correlation between node bridge expected influences in the full sample and subsample with the red area depicting the 2.5th quantile to the 97.5th quantile.
